# Arthroscopic Removal of Suprapatellar Fibroma of Tendon Sheath

**DOI:** 10.1055/s-0037-1601368

**Published:** 2017-03-30

**Authors:** Nan Lou, Christian Fang, Frankie Leung, Florence Cheung, Tak Man Wong

**Affiliations:** 1 Department of Orthopaedics and Traumatology, The University of Hong Kong-Shenzhen Hospital, Shenzhen, China; 2 Department of Orthopaedics and Traumatology, The University of Hong Kong, Queen Mary Hospital, Hong Kong; 3 The University of Hong Kong-Shenzhen Hospital-Shenzhen Key Laboratory for Innovative Technology in Orthopaedic Trauma, Shenzhen, China; 4 Department of Pathology, The University of Hong Kong-Shenzhen Hospital, Shenzhen, China

**Keywords:** intra-articular fibroma, fibroma of tendon sheath, knee joint, arthroscopy

## Abstract

Intra-articular fibroma of tendon sheath is a rare disease. To our knowledge, less than 20 cases have been reported in the literature, and none of them was a Chinese patient. In this case report, we present a Chinese patient with intra-articular fibroma of tendon sheath of the knee joint which was excised arthroscopically. We also summarized the clinical presentation, diagnosis, and subsequent management of intra-articular fibroma of tendon sheath.

Fibroma of tendon sheath (FTS) is a rare dense fibrous benign tumor that majorities are found over the tendons or tendon sheaths of limbs, with predilection in order starting with fingers, hands, wrists, and other parts. 1 Almost 80% of tendon sheath fibromas are found over hands and wrists. 2 The typical morphological feature is a solid nodule that is painless and slow growing in nature. Fibromas of tendon sheath rarely arise from knee joints. To our best knowledge, less than 20 such cases have been reported, and none of them was a Chinese patient. We present a case of intra-articular fibroma of tendon sheath of the knee that was excised arthroscopically.

## Case Report

A 36-year-old male patient had complained a painless mass over the suprapatellar region of left knee for 2 months. He did not complain any systemic upset, and he did not recall any trauma history. Physical examination showed a palpable mass over a suprapatellar region of left knee, which was mobile and firm. The range of motion of the left knee was from 0 to 110 degrees. The joint line was not tender. All special tests including McMurray test, Lachman test, and grinding test of patella were negative.

**Fig. 1:**
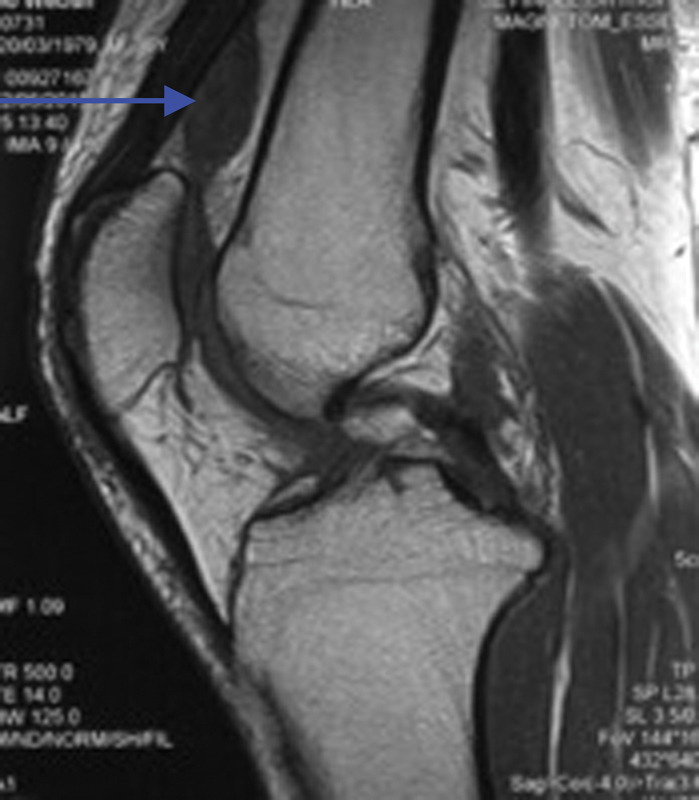
Suprapatellar mass which was hypointense in T1-weighted image (blue arrow).

**Fig. 2:**
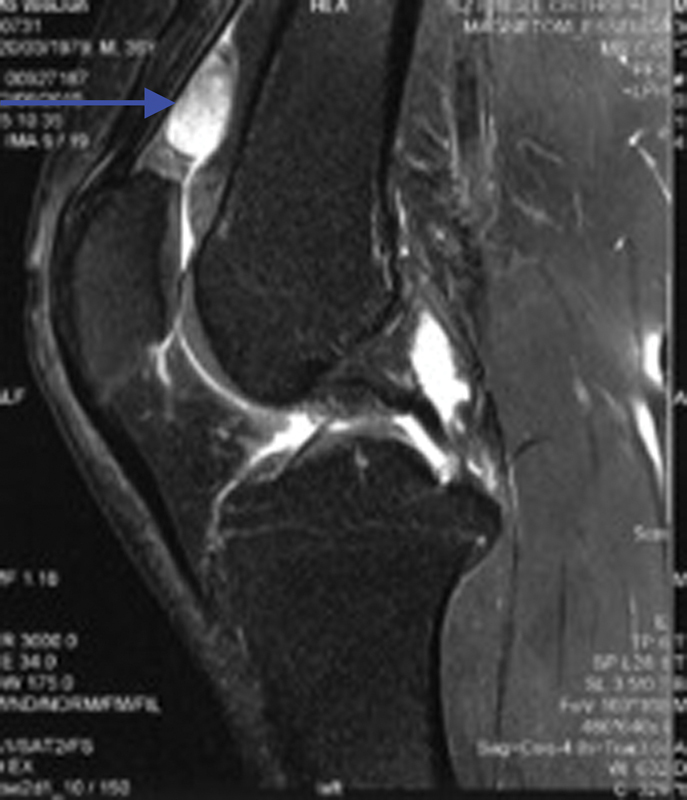
The mass was hyperintense in T2-weighted image (blue arrow).

X-ray examination of the knee showed no bony abnormality. Preoperative magnetic resonance imaging (MRI) of the left knee showed a suprapatellar mass measured 3 cm × 2 cm, hypointense in T1-weighted image ( Fig. 1 ) but hyperintense in T2-weighted image ( Fig. 2 ).

**Fig. 3:**
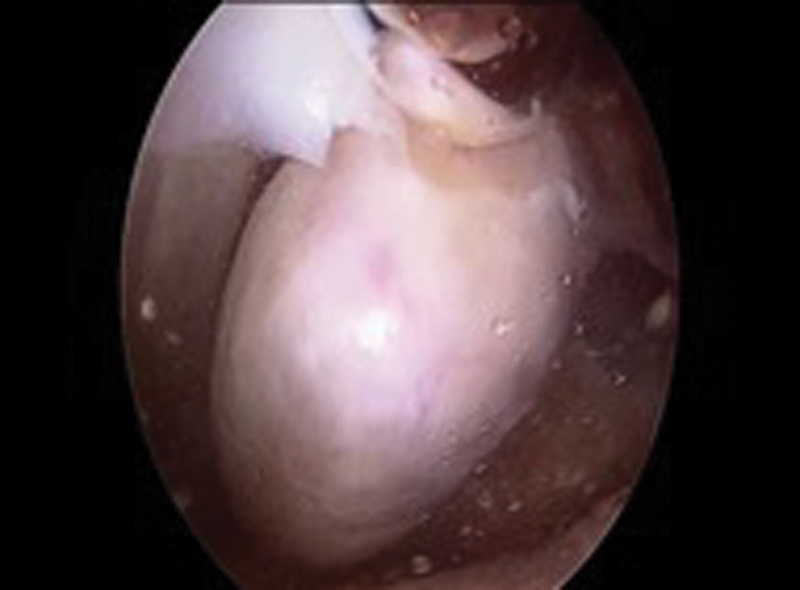
Arthroscopy of left knee showed suprapatellar mass which was grayish and circumferential in appearance.

**Fig. 4:**
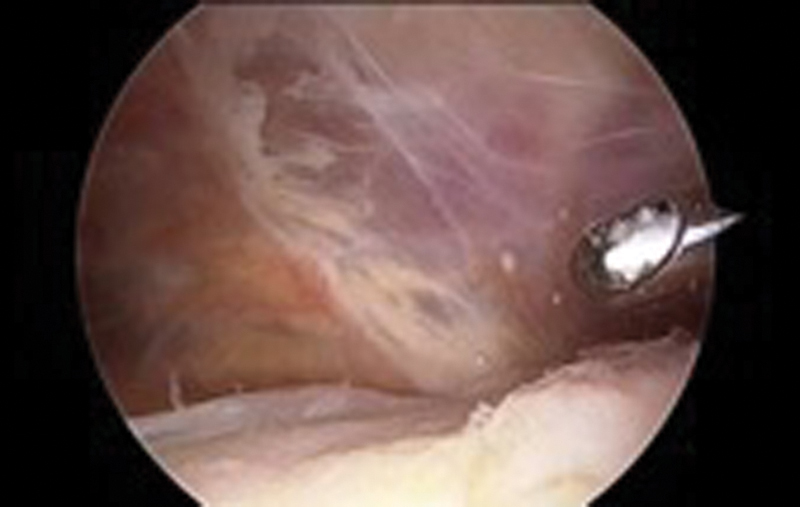
The tumor was excised arthroscopically.

**Fig. 5:**
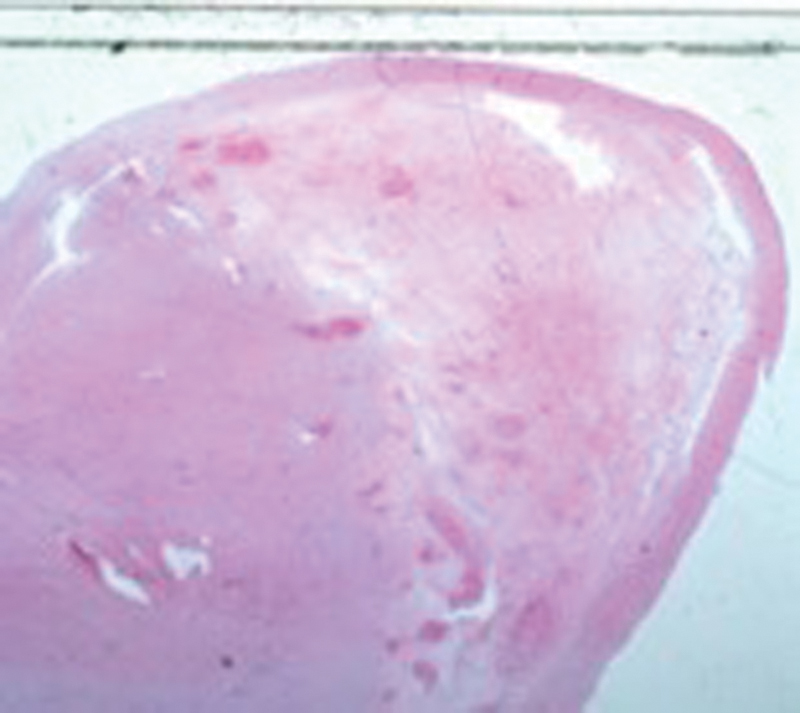
Oval-shaped tumor covered by synovium on one side (hematoxylin and eosin section, ×5).

**Fig. 6:**
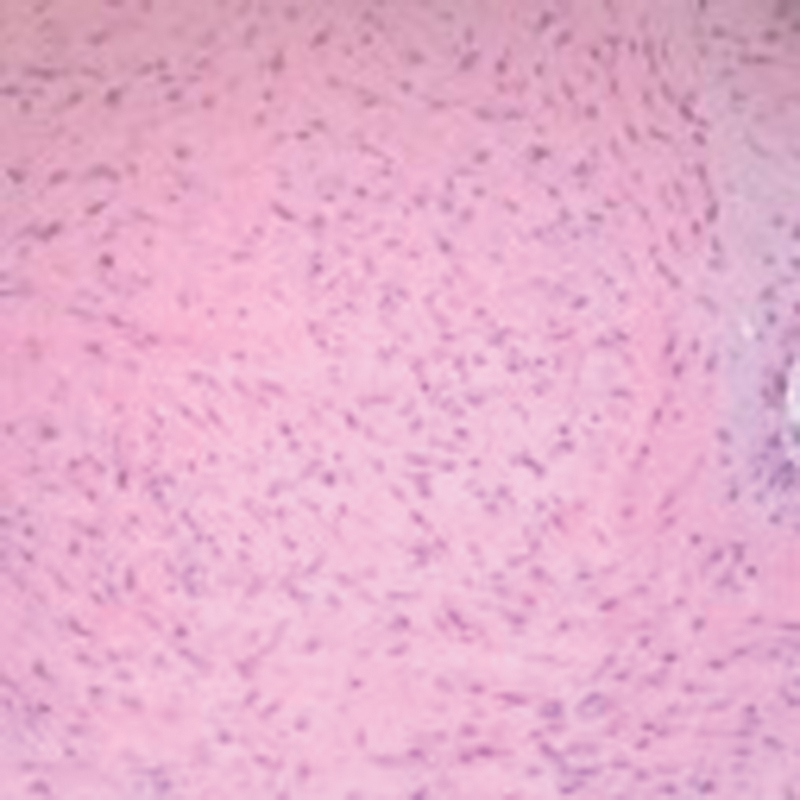
Bland-looking spindle cells proliferating in fibrous stroma (hematoxylin and eosin section, × 200)

**Fig. 7:**
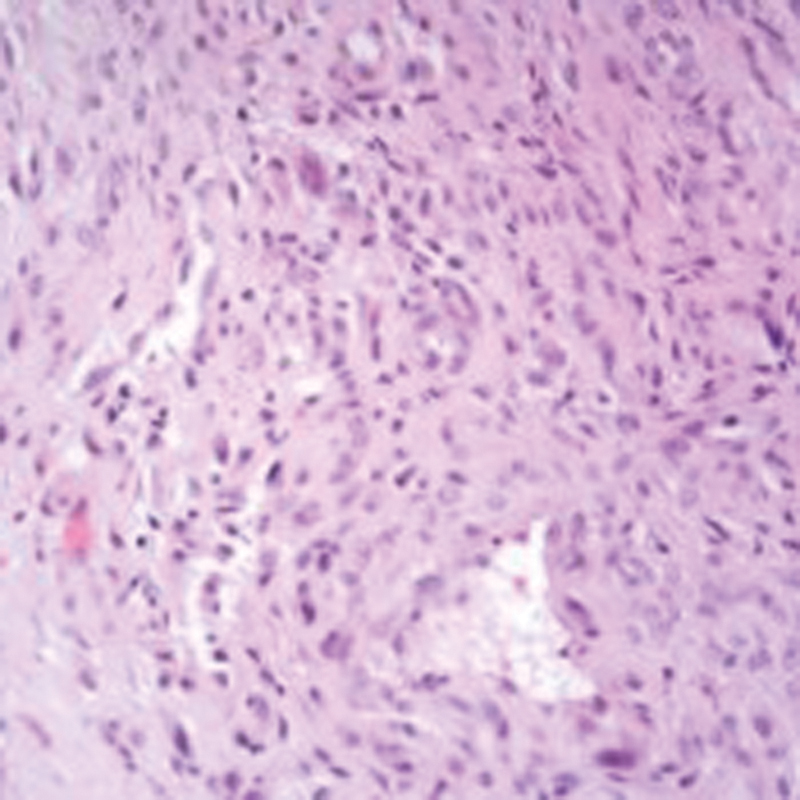
The cellular areas with scanty multinucleated giant cells and rare mitosis (hematoxylin and eosin section, ×400).

Arthroscopic examination of left knee was performed under spinal anesthesia. The tumor was located at the suprapatellar region as shown in MRI. The isolated circumferential mass was grayish, and there was a stalk attaching to the superior pole of the patella ( Fig. 3 ). The tumor was excised through anterolateral and anteromedial portals ( Fig. 4 ). Pathology showed that the mass was covered by synovium on one side with the solid grayish white cut surface ( Fig. 5 ). Histology confirmed a bland-looking spindle-celled neoplasm with infarctive necrosis at the subsynovial region ( Fig. 6 ). The tumor had low cellularity, cleft-like vascular spaces and fibrous stroma in most areas. Cellularity increased toward the root of the tumor with rare typical mitoses. Scanty multinucleated giant cells were seen ( Fig. 7 ). Immunohistochemical studies showed that the spindle cells were focally positive for smooth muscle actin, negative for desmin, and CD34. Proliferation by Ki67 was approximately 10%. Overall features were compatible with intra-articular fibroma of synovial origin. The patient was reassessed at 6 months' time and did not complain of any symptom.

## Discussion

FTS is most commonly found in adults, peaking at 30 to 50 years old and males are commonly affected. 1 3 Since Chung and Enzinger had reported 138 cases of FTS, the histological characteristics and clinical manifestation have become clear. However, it is still difficult to define whether FTS is a reactive fibrosis or a neoplasm. Dal Cin et al reported that fibroma of tendon sheath is a kind of neoplasm rather than reactive fibrosing process. 4 The most common sites of FTS occurrence are upper limbs, especially in the fingers, hands, and wrist, which account for 80 to 86% of all cases. 2 The less common sites of occurrence are toe, foot, ankle, knee, temporomandibular joint, shoulder, and back. FTS is a slow-growing tumor, and most patients complained of a slow-growing painless mass. 5 Less than 10% of patients has history of trauma. 2

X-rays are usually normal, except the tumor is very large compressing the surrounding bony tissue causing erosion. 6 MRI has greatly enhanced ability to display the details of the lesion, but the features are variable. The majority of FTS lesions are hypointense in T1-weighted images, but variable in T2-weighted images, either low signal, high signal, or even mixed. Pinar et al believed that the variability of T2-weighted images signal is associated with the extent of tumor hyaline degeneration and number of fibroblast cells. 7 Giant cell tumor and pigmented villonodular synovitis(PVNS) should be considered when such images are encountered. Giant cell tumor (GCT) is usually isointense and hyperintense in T1-weighted, and T2-weighted images, respectively, while pigmented villonodular synovitis is hypointense in both T1- and T2-weighted images. Apart from GCT and PVNS, another differential diagnosis included nodular fasciitis(NF) 1 and extra-abdominal desmoid tumor. Histogically, NF is similar to FTS, but FTS is more hypocellular and densely collagenous than NF. In our study, the T2-weighted images showed hyperintense signal in which there was extensive hyaline degeneration. Most FTSs are lobulated, firm, and gray in appearance. 8 9 In histological examination, typical FTS has a rich fibroblastic stroma, which is bounded by dense collagen fibers. Leaflets are formed by sparsely scattered fibroblasts, a large number of dense stained eosinophilic collagen fibers and a narrow gap of blood vessels, which are important morphological features of FTS. 1 Treatment of intra-articular fibroma includes either open excision or arthroscopic excision. However, due to the relatively high recurrence rate around 24%, 1 regular follow-up with interval imaging is recommended. In our case, the patient did not have any complaint at 6 months follow-up. An longer period of follow-up would be required to ascertain no recurrence.

## Conclusion

In conclusion, intra-articular FTS is a rare disease, but it should be considered as one of the differential diagnosis of a soft tissue tumor arising from the knee joint. MRI is necessary for diagnosis of intra-articular FTS. Simple excision either open approach or arthroscopy can cure the disease. Regular follow-up with interval imaging is recommended as recurrence rate is relative high.
